# DNA barcoding of the genus *Nepenthes* (Pitcher plant): a preliminary assessment towards its identification

**DOI:** 10.1186/s12870-018-1375-5

**Published:** 2018-08-03

**Authors:** Barbi Gogoi, Brijmohan Singh Bhau

**Affiliations:** 1 Plant Genomics Laboratory, Medicinal Aromatic, and Economic Plant Group, Biological Science and Technology Division, CSIR-North-East Institute of Science & Technology, Jorhat, Assam 785006 India; 2 Academy of Scientific and Innovative Research (AcSIR), CSIR-North-East Institute of Science & Technology, Jorhat, Assam 785006 India; 3 Department of Botany, Central University of Jammu, Rahya-Suchani (Bagla), District – Samba-181 143, Jammu, Jammu & Kashmir India

**Keywords:** *Nepenthes*, Pitcher plant, DNA barcoding, Phylogenetic, ITS, Taxonomy

## Abstract

**Background:**

DNA barcoding is impending towards the generation of universal standards for species discrimination with a standard gene region that can be sequenced accurately and within short span of time. In this study, we were successful in developing efficient barcode locus in the *Nepenthes* genus. A total of 317 accessions were retrieved from GenBank of NCBI which represent 140 different species *Nepenthes* and evaluated the efficacy of ITS, rbcl and matK barcode candidates using barcode gap, applied distance similarity, and tree-based methods.

**Result:**

Our result indicates that single-locus ITS or combined with plastid regions (matK) showed the best species discrimination with distinctive barcoding gaps. Therefore, we tentatively proposed the combination of ITS+matK as a core barcode for *Nepenthes* genus.

**Conclusion:**

This study provides a report on DNA barcoding for unique insectivores’ *Nepenthes* genus. As the different species of *Nepenthes* are higly endemic and endangered, it would be a useful study to understand the evolutionary relationship, sketched in emigration, mislabeling and can be a probable assessment for its biodiversity.

**Electronic supplementary material:**

The online version of this article (10.1186/s12870-018-1375-5) contains supplementary material, which is available to authorized users.

## Background

Taxonomy is the fundamental base for exact nomenclature of a species in an ecosystem. The knowledge gap in taxonomy is increasing due to inadequate taxonomic experts and till today millions of species are still unidentified without proper genetic and biological distribution. Therefore, it is an urgent requirement for definite classification and taxonomy of various delineated species for many theoretical studies and realistic applications [1]. Traditionally morphology-based taxonomy provides ambiguous phylogenetic evidence of large diversified plants genera [2]. To overcome this problem in taxonomy, sequencing of genomic DNA can serve as a standardized method for species identification since, more closely related species hold more homologous DNA sequences in contrast to the distantly associated species [3]. DNA barcoding is regarded as a promising method for proper identification of species using short region of specific DNA sequence efficiently [2, 4]. In animal genomes, mitochondrial cytochrome oxidase I (COI) gene is universally accepted DNA barcode while this region in plants shows insufficient variability caused by its low mutation rate and hence requiring alternative barcoding regions [5–7]. As a result, several chloroplast loci and combinations of these loci have been proposed as a promising DNA barcode in plants [8]. In addition to plastid DNA sequence, nuclear ribosomal internal transcribed spacer (ITS) region is also being used in plants [9, 10]. However, it endures complications in amplification that render it feasibility as a universal barcode for land plants. Despite these complications, many researchers proved that ITS can perform better amplification when compared to other coding or non-coding plastids markers [11–13]. As limited research is carried out in different genera of angiosperm and *Nepenthes* being one of the highly endangered genus, so it is imperative to study about its taxonomic classification and diversity.

*Nepenthes* (Caryophyllales: Nepenthaceae), which includes 170 species around the world, ranging from northern Australia throughout South-east Asia to southern China [14] and New Caledonia and extending westwards to Seychelles and Malagasy. They exhibit a vast diversity in its growth forms, habitats, prey spectra and pitcher form. *Nepenthes sp*. protected under Law no. 5 (1990) on Conservation of Biological Resources and Ecosystem and lined with the regulations of the Convention on International Trade in Endangered Species (CITES) where *N. rajah* and *N. khasiana* are listed on Appendix-I and the rest in APPENDIX-II [15, 16]. This makes the trading activity restricted for this genus. Human interest in *Nepenthes* ranges from the utilization to its therapeutic efficacy. Its unique features of habitat and varied pitcher forms made the genus as an object of fascination and fashionable towards the mankind. Moreover, the highly slippery wax surfaces of the pitcher interior also encouraged engineers to develop many unique products based on this feature. The population of this genus is declining rapidly due to overexploitation and if such declination continues then it will lead to decrease in diversity and result into its extinction. The taxonomy of *Nepenthes* is primarily based on morphology such as shape, color, size and ornamentation [17, 18]. The record on the botanical history of *Nepenthes* showed that there were various cases of taxonomic confusion such as *N. pilosa* with *N. chaniana* until 2006, similarly *N. talangensis* with *N. bongso* and *N. lamii* with *N. vieillardii* [18, 19]. In addition to this, the evolution of genus is challenging as they have no close relatives/ancestral types or transitional species. But *Nepenthes* have distant relatives which can provide a clue about the origin of the genus. Previously, molecular phylogenetic studies in *Nepenthes* were based on chloroplast (trnK and matK gene) and nuclear (PRT1) sequences [20, 21]; however recent studies are based on molecular markers like RAPD, ISSR, etc. [22, 23]. The applicability and effectiveness of DNA barcoding in discriminating the species of *Nepenthes* were conducted for the first time in this study. On the other hand, it is difficult to collect all the species of this genus throughout the large geographical regions. So, this study focuses on the sequences of *Nepenthes* species which are reported in the National centre for Biotechnology Information (NCBI) database. Here, we assessed three potential barcodes by sampling 140 species of *Nepenthes* with the aims of proposing a practical and universal standard barcode region that must be conserved and distinguish the species from the other genera.

## Methods

### Taxon sampling

The loci of ITS, rbcl and matK were selected as barcode candidates in this study. All the available sequences of *Nepenthes* were downloaded from GenBank of NCBI. The sequences were chosen based on two criteria: i. appropriate voucher specimens, and ii. more than 300 bp in length. The taxa, authors and GenBank accession numbers used in this study are shown in Additional file 1: Table S1.

### Data analysis

The downloaded sequences for each region were aligned using Clustal Xv1.8.7 [24] and synchronized manually in BioEdit v7.1.3.0 [25]. For ITS, we adjusted the regions (ITS1 and ITS2) in two ends of 5.8S rDNA based on parsimony principle [14]. Parsimony principle states that in a given set of possible explanation, the simplest explanations are expected to be accurate. On the basis of phylogeny, parsimony means hypothesis of relationships in which least number of character changes is considered most likely to be correct. Hence, all the ITS sequences were aligned and arranged based on parsimony principle in order to avoid erroneous results.

The genetic pair wise distance was computed with Kimura-2-parameter (K2P) distance in MEGA 7. K2P is one of the optimal models for very small distances [2]. The differences between intra- and inter-specific distances for each pair of three single barcodes were compared using pair wise distance in MEGA 7 software. Barcoding gap is the measure of effective barcode locus that exists when the minimum K2P interspecific distance is larger than the maximum intraspecific distance [26]. Taxon DNA with ‘pairwise summary function’ was used to estimate the barcoding gap comparing the distributions of the pairwise intra- and inter-specific distance for each barcode candidate with an interval distance of 0.05.

In order to analyze the species accurately, each barcode candidate was measured for correct identification proportion using Taxon DNA with Best match, ‘Best close match’ and ‘all species barcodes functions. The ‘Best match’ analyses determine the closest match for a given sequence. If the compared sequences were from the same species then the identification is considered as correct whereas incorrect if the sequences did not belong to the same species [27].

To access the effectiveness of marker discriminatory performance, we evaluated the origin of monophyletic by conducting tree-based analysis [26, 28, 29]. The phylogenetic trees were estimated using Neighbor-joining (NJ) in MEGA 7, and node support was assessed by a bootstrap test [30] with 1000 pseudo-replicates of run with the K2P distance as a model of substitution. *Triphyophyllum peltatum* was used as an outgroup.

## Results

Based on the two criteria of screening sequences, we obtained 317 sequences from NCBI, which include 183, 33 and 101 sequences of ITS, rbcl and matK, respectively (Additional file 1: Table S1).

### Genetic divergence analysis

The aligned sequence lengths ranged from 1251 bp for rbcl to 951 bp for ITS (Table 1).ITS had the maximum variable sites and parsimony-informative characters followed by matK. The intra-specific distance in the six barcodes ranged from 0.0 to 0.9% and the mean intra-specific distances were least for rbcl+matK (0.02%) and highest for ITS (1.31%). Subsequently, the pairwise inter-specific distances were ranged from 0.0 to 1.18% and the mean inter-specific distance was minimum for ITS+rbcl (0.16%) and maximum for ITS (0.84%). In summary, ITS reveal the highest mean intra- and inter-specific distances (Table 2).

**Table 1:** Evaluation of three barcoding loci and its combination

	ITS	rbcl	matK	ITS+ rbcl	ITS+ matK	Rbcl+ matK
No. of species samples (individuals)	88 (183)	17 (33)	35 (101)	8 (18)	24 (78)	15 (18)
Aligned sequence length (bp)	948	1251	1136	2114	2101	2417
No. of variable sites	666	282	591	430	957	864
No. of parsimony informative sites	447	269	372	336	685	675
Ability to discriminate (%)	30.68	11.76	22.85	50.00	83.33	13.33

**Table 2:** Summary of the pairwise intra-specific and inter-specific distances in the barcode loci of *Nepenthes* species

Barcode locus	Intra-specific distances (%)			Inter-specific distances (%)		
	Minimum	Maximum	Mean	Minimum	Maximum	Mean
ITS	0	0.16	1.31	0	1.18	0.84
Rbcl	0	0.60	0.22	0	0.33	0.21
MatK	0	0.90	0.54	0	1.15	0.64
ITS+ rbcl	0	0.03	0.12	0	0.59	0.16
ITS+ MatK	0	0.29	0.33	0	0.54	0.22
Rbcl+ MatK	0	0.67	0.02	0	0.69	0.18

### Barcoding gap analysis

**Fig. 1:**
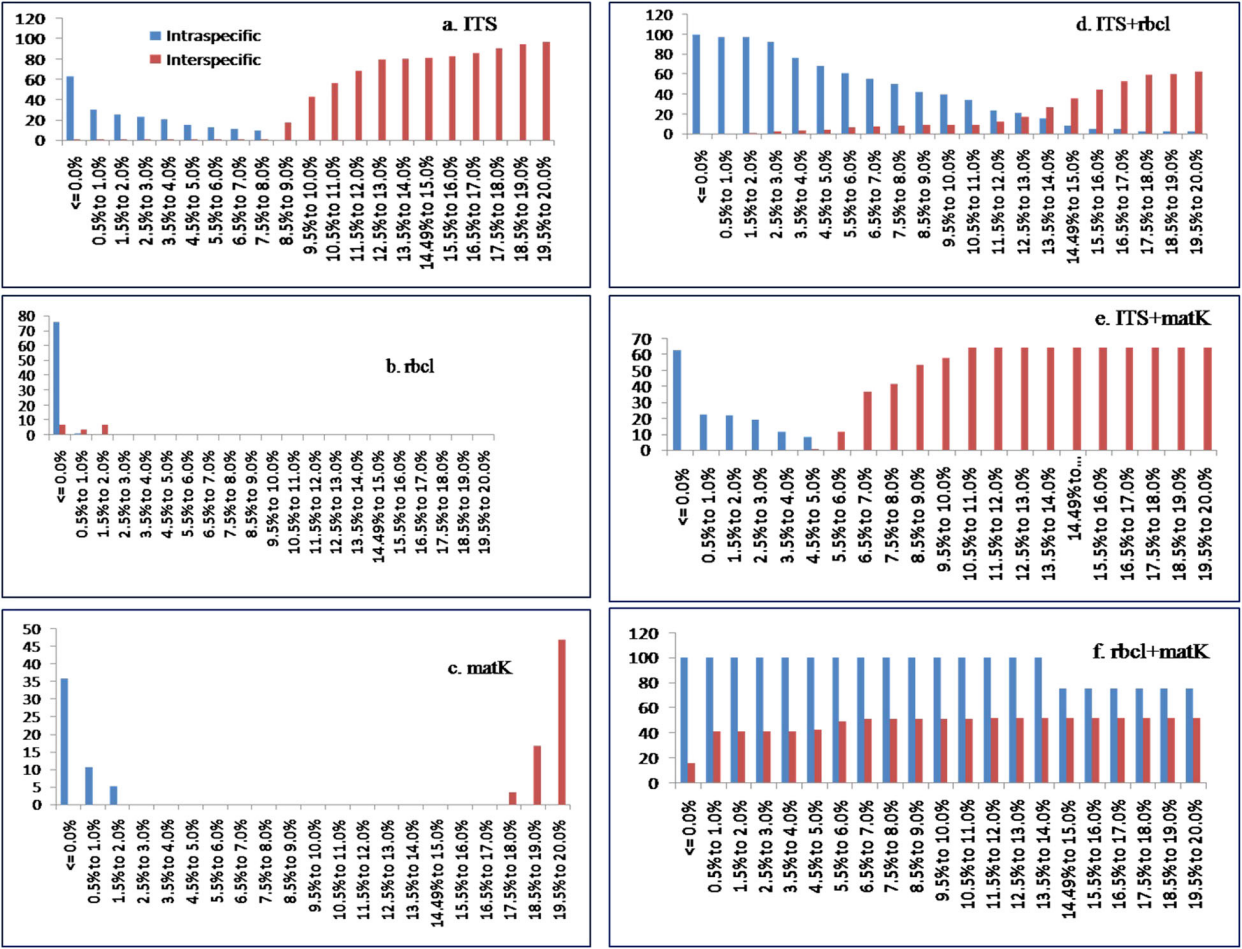
Distribution of intra- and inter-specific Kimura 2-parameter (K2P) distances among all *Nepenthes* samples for the three barcodes loci and their combinations

The relative distribution of barcoding gap between intra- and inter-specific genetic distances were calculated using K2P distances in Taxon DNA software for three barcode candidates. The inter-specific distances were higher in all subgenera and did not fully overlap with intra-specific distance. Therefore, we analyzed barcoding gap for all datasets and subgenera. Three barcodes i.e. ITS (Fig. 1a), matK (Fig. 1c) and ITS+matK (Fig. 1e) showed relatively clear barcoding gaps. All other barcodes had overlapped between their intra- and inter- specific distances without clear barcoding gaps (Fig. 1).

### Discrimination of species

Analysis of discriminating species was performed using Taxon DNA, ITS had the highest success rate for correct identification of species (Best match: 78.12%; Best close match: 77.67%; All species barcodes: 80.76%) followed by ITS+matK and least discrimination success rate was observed in ITS+rbcl (Table 3).

**Table 3:** Identification success based on the ‘best match’, ‘best close match’ and ‘all species barcodes’ function of the program TaxonDNA

Region	Best match			Best Close match			All species barcodes		
	Correct (%)	Ambiguous (%)	Incorrect (%)	Correct (%)	Ambiguous (%)	Incorrect (%)	Correct (%)	Ambiguous (%)	Incorrect (%)
ITS	78.12	30.21	8.46	77.67	29.67	34.06	80.76	7.14	5.49
rbcl	18.75	31.31	3.12	18.75	78.12	10.0	46.87	46.87	3.12
matK	59.4	29.7	10.89	57.42	29.7	10.89	52.47	43.56	1.98
ITS+ rbcl	66.66	7.4	33.33	27.77	1.23	22.22	30.23	44.44	5.55
ITS+ matK	75.38	8.97	25.64	72.82	8.97	12.82	65.38	19.23	1.8
rbcl+ matK	17.69	50.0	50.0	12.43	44.44	16.66	47.50	61.11	1.0

### Tree-based analysis

**Fig. 2:**
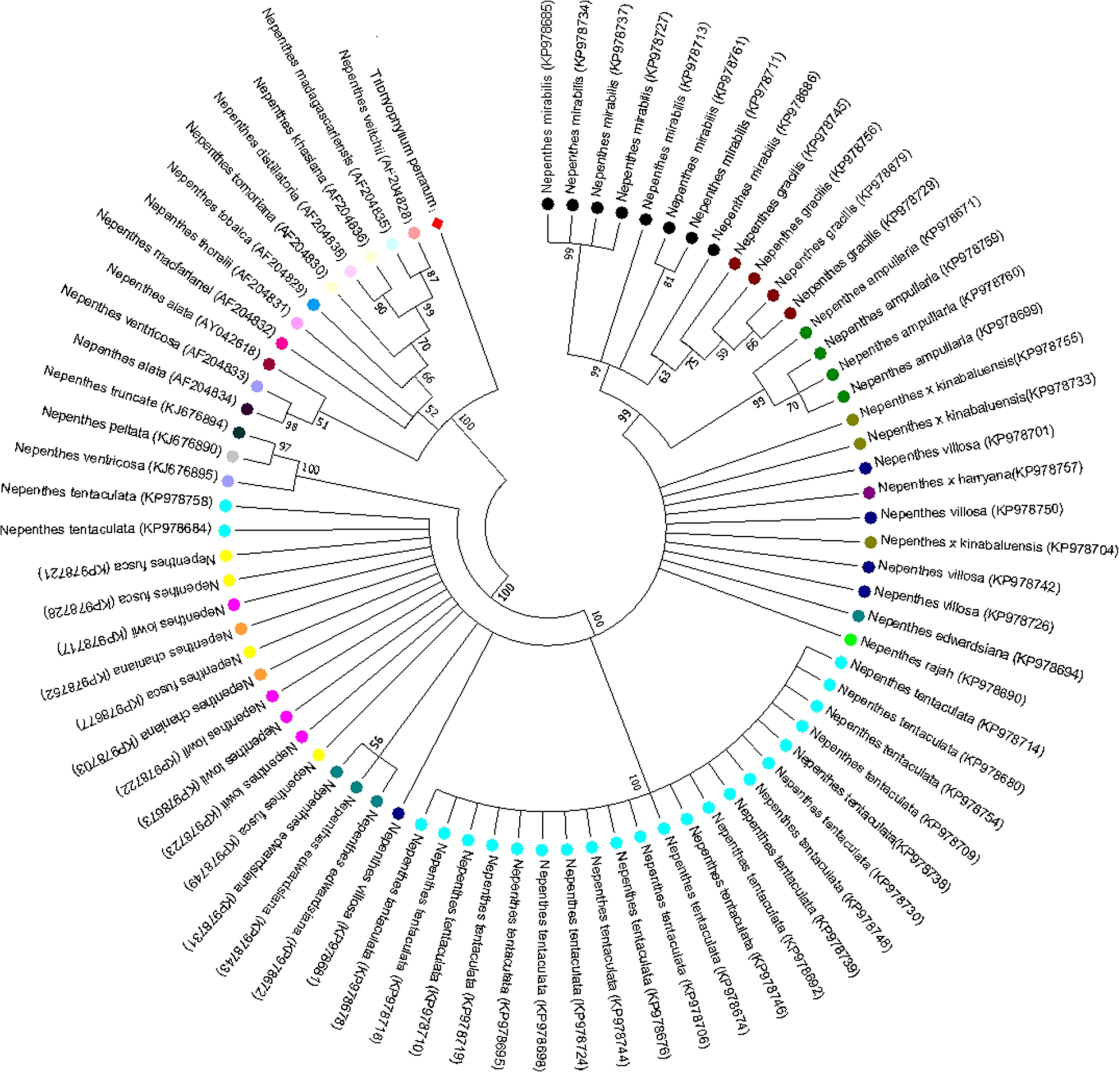
Neighbor joining (NJ) tree generated using ITS+matK sequences of *Nepenthes*. Bootstrap values (> 50%) are shown above the relevant branches. Corresponding different species of *Nepenthes are colour coded*

Discriminating sequences of six barcode candidates based on phylogenetic trees were estimated by evaluating the percentage of each species or variety as well as determined to be monophyletic using NJ tree based analysis (Fig. 2). We observed that all single-locus barcodes had low levels of species discrimination varying from 11.76 to 30.68% (Table 1). Among the multilocus barcodes, ITS+matK showed the maximum success rate (83.33%) followed by ITS+rbcl (50.00%). Thus, it can be concluded that species discrimination was higher when ITS was included among three combinations. We accomplished that our result suggests that ITS+matk is preeminent among all the core barcodes.

## Discussion

Several studies were carried out to discover suitable barcodes for different plants but the desired consensus was achieved so far [31, 32]. In the present study, we included *Nepenthes sp*. sequences obtained from different studies through their GenBank records. Thus, we strongly assumed that all reported sequences of *Nepenthes sp*. were based on correctly identified plant species. Plastids region were initially proposed as core barcode in plants, but they are not successful in all genus of plants. Moreover, many researchers found ITS as a challenging barcode in plants and thus rejected for incorporation in the core barcode region of plants [9, 33–35]. With advanced researches, we observed that the region of ITS was widely used for recovering high rates of correctly assigned species as it posses less intra-specific variation but higher inter-specific divergence [36]. Moreover, the combinations of ITS and plastids loci were found to be the best option in some plant genus. According to our results, ITS and matK had better parsimony informative sites and discriminating power among the proposed barcode loci i.e. ITS, rbcl and matK which relate similarly to the results of previous studies [14, 37, 38]. Discriminating species on the basis of pairwise distances are subjected to be prolific if the inter-specific distances are greater than intra-specific distances [8] and finally we observed that ITS had the highest intra- and inter-specific sequence divergence based on distance analysis methods. The statistics of “best match”, “best close match” and “all species barcodes” options were used in this study and ITS was again observed with high species discrimination rate followed by ITS+MatK. Based on NJ tree, ITS+matK barcode posse’s maximum and rbcl contain minimum species resolution rate for the genus. On the other hand, several combinations of two or three barcodes are being proposed as core barcodes in plants, including ITS+trnH-psbA [12], ITS+rbcl [39], matK+rbcl [8] and ITS+matK+rbcl [28] but a consensus regarding its utility has not been achieved yet. matK+rbcl was considered as an universal barcode for all land plants but in *Nepenthes* sp. matK+rbcl posses low species resolution among the three barcode combinations because of low substitution rates in coding genes where ITS+matK posses the highest percent of species identification as compared to the other single or combinations of barcode candidates which posses well-defined barcode gaps. However, all species of *Nepenthes* are specific or restricted to different geographical regions. Therefore, a potential solution of identifying the species from illegal transfer and geographical information could be achieved with the application of DNA barcoding. In future, these findings will potentially be helpful in delineating the species of *Nepenthes* and hence, they could most likely be successful as barcodes for this genus.

## Conclusion

The present study evaluates DNA barcoding technique for the taxonomic origin/identification of endangered and endemic plants which are illegally traded. From this study, we can conclude that DNA barcode identification can be made more authentic by relying on integrated approach including prior and a posteriori date. In this study, it depicts that among the six barcode candidates single locus ITS and multiple locus ITS+matK posses high rate of discriminating power which can be further accessed as core barcode for Nepenthes genus. As this genus is unique in different parts of the world, an irrefragable system like DNA barcoding is required for conservation in biodiversity and control in the illegal trade of the species.

## Additional file

Additional file 1: **Table S1.** List of samples information for the Nepenthes species used in this study. (PDF 255 kb)
