# Supplementary material for: DNA barcoding of the genus Nepenthes (Pitcher plant): a preliminary assessment towards its identification
Source: BMC Plant Biol. 2018 Aug 3;18:153. doi: 10.1186/s12870-018-1375-5 (PMC6091102; doi:10.1186/s12870-018-1375-5)
Supplement: Supplementary file 1 — Table S1. List of samples information for the Nepenthes species used in this study. (PDF 255 kb) [file 12870_2018_1375_MOESM1_ESM.pdf]

Table S1. List of samples information for the *Nepenthes* species used in this study.

Sl No.	Taxon	Author	Accession no.
**ITS**			
1	*Nepenthes alata*	Renner,T. *et al.*	HM204891
2	*Nepenthes alata*	Alejandro,G.D. *et al.*,	AM269504
3	*Nepenthes alba*	Bunawan,H. *et al.*	JX042564
4	*Nepenthes albomarginata*	Renner,T. *et al.*	HM204892
5	*Nepenthes albomarginata*	Bunawan,H. *et al.*	JX042559
6	*Nepenthes andamana*	Merckx,V.S. *et al.*	KR698380
7	*Nepenthes ampullaria*	Bunawan,H. *et al.*	JX042554
8	*Nepenthes ampullaria*	Merckx,V.S. *et al.*	KP978817
9	*Nepenthes ampullaria*	Merckx,V.S. *et al.*	KP978925
10	*Nepenthes ampullaria*	Merckx,V.S. *et al.*	KP978924
11	*Nepenthes ampullaria*	Merckx,V.S. *et al.*	KP978762
12	*Nepenthes ampullaria*	Alamsyah,F. *et al.*	AB675914
13	*Nepenthes beccariana*	Alamsyah,F. *et al.*	AB675709
14	*Nepenthes benstonei*	Alamsyah,F. *et al.*	AB675710
15	*Nepenthes benstonei*	Bunawan,H. *et al.*	JX042560
16	*Nepenthes bokorensis*	Merckx,V.S. *et al.*	KR698372
17	*Nepenthes bongso*	Schwallier,R.M. *et al.*	KM982416
18	*Nepenthes bongso*	Alamsyah,F. *et al.*	AB675703
19	*Nepenthes boschiana*	Renner,T. *et al.*	HM204893
20	*Nepenthes campanulata*	Merckx,V.S. *et al.*	KR698373
21	*Nepenthes chaniana*	Schwallier,R.M. *et al.*	KM982417
22	*Nepenthes chaniana*	Merckx,V.S. *et al.*	KP978905
23	*Nepenthes chaniana*	Merckx,V.S. *et al.*	KP978823
24	*Nepenthes danseri*	Alamsyah,F. *et al.*	AB675915
25	*Nepenthes densiflora*	Alamsyah,F. *et al.*	AB675875
26	*Nepenthes diatas*	Alamsyah,F. *et al.*	AB675876
27	*Nepenthes distillatoria*	Alamsyah,F. *et al.*	AB675877
28	*Nepenthes dubia*	Alamsyah,F. *et al.*	AB675698
29	*Nepenthes edwardsiana*	Merckx,V.S. *et al.*	KP978895
30	*Nepenthes edwardsiana*	Merckx,V.S. *et al.*	KP978873
31	*Nepenthes edwardsiana*	Merckx,V.S. *et al.*	KP978811
32	*Nepenthes edwardsiana*	Merckx,V.S. *et al.*	KP978764
33	*Nepenthes ephippiata*	Alamsyah,F. *et al.*	AB675878
34	*Nepenthes eustachya*	Alamsyah,F. *et al.*	AB675702
35	*Nepenthes eymae*	Alamsyah,F. *et al.*	AB675696
36	*Nepenthes faizaliana*	Alamsyah,F. *et al.*	AB675879
37	*Nepenthes fusca*	Renner,T. *et al.*	HM204894
38	*Nepenthes fusca*	Merckx,V.S. *et al.*	KP978901
39	*Nepenthes fusca*	Merckx,V.S. *et al.*	KP978867
40	*Nepenthes fusca*	Merckx,V.S. *et al.*	KP978856
41	*Nepenthes fusca*	Merckx,V.S. *et al.*	KP978771

42	*Nepenthes fusca*	Alamsyah,F. *et al.*	AB675880
43	*Nepenthes glabrata*	Alamsyah,F. *et al.*	AB675881
44	*Nepenthes glandulifera*	Renner,T. *et al.*	HM204895
45	*Nepenthes gracilis*	Bunawan,H. *et al.*	JX042555
46	*Nepenthes gracilis*	Merckx,V.S. *et al.*	KP978853
47	*Nepenthes gracilis*	Merckx,V.S. *et al.*	KP978789
48	*Nepenthes gracilis*	Alamsyah,F. *et al.*	AB675882
49	*Nepenthes gracillima*	Renner,T. *et al.*	HM204896
50	*Nepenthes gymnamphora*	Renner,T. *et al.*	HM204897
51	*Nepenthes gymnamphora*	Alamsyah,F. *et al.*	AB675694
52	*Nepenthes hirsuta*	Alamsyah,F. *et al.*	AB675916
53	*Nepenthes hurrelliana*	Merckx,V.S. *et al.*	KR698374
54	*Nepenthes hurrelliana*	Schwallier,R.M. *et al.*	KM982418
55	*Nepenthes inermis*	Alamsyah,F. *et al.*	AB675701
56	*Nepenthes insignis*	Renner,T. *et al.*	HM204898
57	*Nepenthes insignis*	Merckx,V.S. *et al.*	KR698375
58	*Nepenthes izumiae*	Alamsyah,F. *et al.*	AB675699
59	*Nepenthes jacquelineae*	Alamsyah,F. *et al.*	AB675705
60	*Nepenthes jamban*	Alamsyah,F. *et al.*	AB675704
61	*Nepenthes kampotiana*	Merckx,V.S. *et al.*	KR698376
62	*Nepenthes khasiana*	Bhau,B. *et al.*	KT735323
63	*Nepenthes khasiana*	Bhau,B. *et al.*	KT735322
64	*Nepenthes khasiana*	Bhau,B. *et al.*	KT735321
65	*Nepenthes khasiana*	Bhau,B. *et al.*	KT735320
66	*Nepenthes khasiana*	Bhau,B. *et al.*	KT735319
67	*Nepenthes khasiana*	Bhau,B. *et al.*	KT735318
68	*Nepenthes khasiana*	Bhau,B. *et al.*	KT735317
69	*Nepenthes khasiana*	Bhau,B. *et al.*	KT735316
70	*Nepenthes khasiana*	Bhau,B. *et al.*	KT735315
71	*Nepenthes khasiana*	Bhau,B. *et al.*	KT354296
72	*Nepenthes khasiana*	Bhau,B. *et al.*	KT354295
73	*Nepenthes khasiana*	Alamsyah,F. *et al.*	AB675883
74	*Nepenthes klossii*	Alamsyah,F. *et al.*	AB675716
75	*Nepenthes kongkandana*	Merckx,V.S. *et al.*	KR698377
76	*Nepenthes lingulata*	Alamsyah,F. *et al.*	AB675884
77	*Nepenthes longifolia*	Alamsyah,F. *et al.*	AB675885
78	*Nepenthes lowii*	Alamsyah,F. *et al.*	AB675695
79	*Nepenthes lowii*	Merckx,V.S. *et al.*	KP978859
80	*Nepenthes lowii*	Merckx,V.S. *et al.*	KP978857
81	*Nepenthes lowii*	Merckx,V.S. *et al.*	KP978852
82	*Nepenthes lowii*	Merckx,V.S. *et al.*	KP978766
83	*Nepenthes macfarlanei*	Renner,T. *et al.*	HM204900
84	*Nepenthes macfarlanei*	Bunawan,H. *et al.*	JX042562
85	*Nepenthes macrovulgaris*	Alamsyah,F. *et al.*	AB675886
86	*Nepenthes madagascariensis*	Alamsyah,F. *et al.*	AB769064

87	*Nepenthes maxima*	Renner,T. *et al.*	HM204901
88	*Nepenthes maxima*	Alamsyah,F. *et al.*	AB675697
89	*Nepenthes merrilliana*	Alamsyah,F. *et al.*	AB675887
90	*Nepenthes mikei*	Alamsyah,F. *et al.*	AB675700
91	*Nepenthes mindanaoensis*	Alamsyah,F. *et al.*	AB675888
92	*Nepenthes mira*	Alamsyah,F. *et al.*	AB675711
93	*Nepenthes mirabilis*	Renner,T. *et al.*	HM204902
94	*Nepenthes mirabilis*	Bunawan,H. *et al.*	JX042556
95	*Nepenthes mirabilis*	Alamsyah,F. *et al.*	AB675714
96	*Nepenthes mirabilis*	Merckx,V.S. *et al.*	KP978842
97	*Nepenthes mirabilis*	Merckx,V.S. *et al.*	KP978821
98	*Nepenthes mirabilis*	Merckx,V.S. *et al.*	KP978795
99	*Nepenthes mirabilis*	Alamsyah,F. *et al.*	AB675890
100	*Nepenthes mirabilis*	Alamsyah,F. *et al.*	AB675889
101	*Nepenthes naga*	Alamsyah,F. *et al.*	AB675891
102	*Nepenthes neoguineensis*	Alamsyah,F. *et al.*	AB675917
103	*Nepenthes northiana*	Renner,T. *et al.*	HM204903
104	*Nepenthes ovata*	Alamsyah,F. *et al.*	AB675892
105	*Nepenthes papuana*	Alamsyah,F. *et al.*	AB675918
106	*Nepenthes pectinata*	Alamsyah,F. *et al.*	AB675708
107	*Nepenthes peltata*	Merckx,V.S. *et al.*	KR698378
108	*Nepenthes petiolata*	Alamsyah,F. *et al.*	AB675713
109	*Nepenthes rafflesiana*	Renner,T. *et al.*	HM204904
110	*Nepenthes rafflesiana*	Bunawan,H. *et al.*	JX042558
111	*Nepenthes rajah*	Merckx,V.S. *et al.*	KP978801
112	*Nepenthes rajah*	Alamsyah,F. *et al.*	AB675895
113	*Nepenthes ramispina*	Bunawan,H. *et al.*	JX042563
114	*Nepenthes reinwardtiana*	Renner,T. *et al.*	HM204905
115	*Nepenthes reinwardtiana*	Alamsyah,F. *et al.*	AB675896
116	*Nepenthes rhombicaulis*	Alamsyah,F. *et al.*	AB675897
117	*Nepenthes rowaniae*	Alamsyah,F. *et al.*	AB675919
118	*Nepenthes sanguinea*	Renner,T. *et al.*	HM204906
119	*Nepenthes sanguinea*	Bunawan,H. *et al.*	JX042561
120	*Nepenthes sanguinea*	Alamsyah,F. *et al.*	AB675898
121	*Nepenthes singalana*	Renner,T. *et al.*	HM204907
122	*Nepenthes smilesii*	Alamsyah,F. *et al.*	AB675899
123	*Nepenthes spathulata*	Alamsyah,F. *et al.*	AB675900
124	*Nepenthes spathulata*	Alamsyah,F *et al.*	AB675693
125	*Nepenthes spectabilis*	Renner,T. *et al.*	HM204908
126	*Nepenthes spectabilis*	Alamsyah,F. *et al.*	AB675901
127	*Nepenthes stenophylla*	Alamsyah,F. *et al.*	AB675903
128	*Nepenthes sumatrana*	Alamsyah,F. *et al.*	AB675904
129	*Nepenthes talangensis*	Alamsyah,F. *et al.*	AB675905
130	*Nepenthes tentaculata*	Renner,T. *et al.*	HM204909
131	*Nepenthes tentaculata*	Merckx,V.S. *et al.*	KP978920

132	*Nepenthes tentaculata*	Merckx,V.S. *et al.*	KP978910
133	*Nepenthes tentaculata*	Merckx,V.S. *et al.*	KP978900
134	*Nepenthes tentaculata*	Merckx,V.S. *et al.*	KP978898
135	*Nepenthes tentaculata*	Merckx,V.S. *et al.*	KP978897
136	*Nepenthes tentaculata*	Merckx,V.S. *et al.*	KP978879
137	*Nepenthes tentaculata*	Merckx,V.S. *et al.*	KP978878
138	*Nepenthes tentaculata*	Merckx,V.S. *et al.*	KP978872
139	*Nepenthes tentaculata*	Merckx,V.S. *et al.*	KP978861
140	*Nepenthes tentaculata*	Merckx,V.S. *et al.*	KP978854
141	*Nepenthes tentaculata*	Merckx,V.S. *et al.*	KP978849
142	*Nepenthes tentaculata*	Merckx,V.S. *et al.*	KP978843
143	*Nepenthes tentaculata*	Merckx,V.S. *et al.*	KP978838
144	*Nepenthes tentaculata*	Merckx,V.S. *et al.*	KP978832
145	*Nepenthes tentaculata*	Merckx,V.S. *et al.*	KP978829
146	*Nepenthes tentaculata*	Merckx,V.S. *et al.*	KP978815
147	*Nepenthes tentaculata*	Merckx,V.S. *et al.*	KP978812
148	*Nepenthes tentaculata*	Merckx,V.S. *et al.*	KP978808
149	*Nepenthes tentaculata*	Merckx,V.S. *et al.*	KP978792
150	*Nepenthes tentaculata*	Merckx,V.S. *et al.*	KP978777
151	*Nepenthes tentaculata*	Merckx,V.S. *et al.*	KP978772
152	*Nepenthes tentaculata*	Merckx,V.S. *et al.*	KP978770
153	*Nepenthes tentaculata*	Merckx,V.S. *et al.*	KP978767
154	*Nepenthes tentaculata*	Merckx,V.S. *et al.*	AB675920
155	*Nepenthes tenuis*	Alamsyah,F. *et al.*	AB675707
156	*Nepenthes thai*	Alamsyah,F. *et al.*	AB675906
157	*Nepenthes thorelii*	Alamsyah,F. *et al.*	AB675712
158	*Nepenthes tobaica*	Alamsyah,F. *et al.*	AB675907
159	*Nepenthes tomoriana*	Alamsyah,F. *et al.*	AB675706
160	*Nepenthes truncata*	Renner,T. *et al.*	HM204910
161	*Nepenthes truncata*	Alamsyah,F. *et al.*	AB675908
162	*Nepenthes veitchii*	Alamsyah,F. *et al.*	AB675909
163	*Nepenthes ventricosa*	Renner,T. *et al.*	HM204911
164	*Nepenthes ventricosa*	Alejandro,G.D.,	AM269503
165	*Nepenthes ventricosa*	Alamsyah,F. *et al.*	AB675910
166	*Nepenthes vieillardii*	Renner,T. *et al.*	HM204912
167	*Nepenthes vieillardii*	Alamsyah,F. *et al.*	AB769065
168	*Nepenthes villosa*	Merckx,V.S. *et al.*	KP978902
169	*Nepenthes villosa*	Merckx,V.S. *et al.*	KP978892
170	*Nepenthes villosa*	Merckx,V.S. *et al.*	KP978864
171	*Nepenthes villosa*	Merckx,V.S. *et al.*	KP978820
172	*Nepenthes villosa*	Merckx,V.S. *et al.*	KP978785
173	*Nepenthes villosa*	Alamsyah,F. *et al.*	AB675911
174	*Nepenthes vogelii*	Schwallier,R.M *et al.*	KM982419
175	*Nepenthes vogelii*	Merckx,V.S. *et al.*	KR698379
176	*Nepenthes vogelii*	Alamsyah,F. *et al.*	AB675912

177	*Nepenthes x harryana*	Merckx,V.S. *et al.*	KP978917
178	*Nepenthes x intermedia*	Renner,T. *et al.*	HM204899
179	*Nepenthes x kinabaluensis*	Merckx,V.S. *et al.*	KP978912
180	*Nepenthes x kinabaluensis*	Merckx,V.S. *et al.*	KP978876
181	*Nepenthes x kinabaluensis*	Merckx,V.S. *et al.*	KP978824
**rbcL**			
182	*Nepenthes alata*	Acil,R.Y. *et al.*	KJ659423
183	*Nepenthes alata*	Acil,R.Y. *et al.*	KM393201
184	*Nepenthes alata*	Acil,R.Y. *et al.*	KM393195
185	*Nepenthes alata*	Albert,V.A. *et al.*	L01936
186	*Nepenthes bellii*	Acil,R.Y. *et al.*	KJ659424
187	*Nepenthes bellii*	Acil,R.Y. *et al.*	KM393196
188	*Nepenthes ceciliae*	Acil,R.Y. *et al.*	KJ659425
189	*Nepenthes copelandii*	Acil,R.Y. *et al.*	KJ659426
190	*Nepenthes hamiguitanensis*	Acil,R.Y. *et al.*	KJ659427
191	*Nepenthes khasiana*	Bhau,B. *et al.*	KT285307
192	*Nepenthes khasiana*	Bhau,B. *et al.*	KT309042
193	*Nepenthes khasiana*	Bhau,B. *et al.*	KT309041
194	*Nepenthes khasiana*	Bhau,B. *et al.*	KT309040
195	*Nepenthes khasiana*	Bhau,B. *et al.*	KT309039
196	*Nepenthes khasiana*	Bhau,B. *et al.*	KT309038
197	*Nepenthes khasiana*	Bhau,B. *et al.*	KT309037
198	*Nepenthes khasiana*	Bhau,B. *et al.*	KT309036
199	*Nepenthes khasiana*	Bhau,B. *et al.*	KT309035
200	*Nepenthes merrilliana*	Acil,R.Y. *et al.*	KJ659428
201	*Nepenthes micramphora*	Acil,R.Y. *et al.*	KJ659429
202	*Nepenthes mindanaoensis*	Acil,R.Y. *et al.*	KJ659430
203	*Nepenthes mindanaoensis*	Acil,R.Y. *et al.*	KM393197
204	*Nepenthes peltata*	Acil,R.Y. *et al.*	KJ659431
205	*Nepenthes peltata*	Acil,R.Y. *et al.*	KM393198
206	*Nepenthes pulchra*	Acil,R.Y. *et al.*	KJ659432
207	*Nepenthes saranganiensis*	Acil,R.Y. *et al.*	KJ659433
208	*Nepenthes surigaoensis*	Acil,R.Y. *et al.*	KJ659434
209	*Nepenthes truncata*	Acil,R.Y. *et al.*	KJ659435
210	*Nepenthes ventricosa*	Acil,R.Y. *et al.*	KJ659436
211	*Nepenthes vieillardii*	Kurata,K. *et al.*	AB103324
212	*Nepenthes vieillardii*	Kurata,K. *et al.*	AB103323
213	*Nepenthes vieillardii*	Kurata,K. *et al.*	AB103322
214	*Nepenthes vieillardii*	Kurata,K. *et al.*	AB103321
215	*Nepenthes vieillardii*	Kurata,K. *et al.*	AB103320
216	*Nepenthes vieillardii*	Kurata,K. *et al.*	AB103319
**matK**			
217	*Nepenthes alata*	Meimberg,H. *et al.*	AF204834

218	*Nepenthes alata*	Cuenoud,P. *et al.*	AY042618
219	*Nepenthes alata*	Acil,R.Y. *et al.*	KJ676882
220	*Nepenthes alata*	Acil,R.Y. *et al.*	KM393199
221	*Nepenthes ampullaria*	Merckx,V.S. *et al.*	KP978671
222	*Nepenthes ampullaria*	Merckx,V.S. *et al.*	KP978760
223	*Nepenthes ampullaria*	Merckx,V.S. *et al.*	KP978759
224	*Nepenthes ampullaria*	Merckx,V.S. *et al.*	KP978699
225	*Nepenthes bellii*	Acil,R.Y. *et al.*	KJ676883
226	*Nepenthes ceciliae*	Acil,R.Y. *et al.*	KJ676884
227	*Nepenthes chaniana*	Merckx,V.S. *et al.*	KP978752
228	*Nepenthes chaniana*	Merckx,V.S. *et al.*	KP978703
229	*Nepenthes copelandii*	Acil,R.Y. *et al.*	KJ676885
230	*Nepenthes distillatoria*	Meimberg,H. *et al.*	AF204838
231	*Nepenthes edwardsiana*	Merckx,V.S. *et al.*	KP978672
232	*Nepenthes edwardsiana*	Merckx,V.S. *et al.*	KP978743
233	*Nepenthes edwardsiana*	Merckx,V.S. *et al.*	KP978731
234	*Nepenthes edwardsiana*	Merckx,V.S. *et al.*	KP978694
235	*Nepenthes fusca*	Merckx,V.S. *et al.*	KP978677
236	*Nepenthes fusca*	Merckx,V.S. *et al.*	KP978749
237	*Nepenthes fusca*	Merckx,V.S. *et al.*	KP978721
238	*Nepenthes fusca*	Merckx,V.S. *et al.*	KP978728
239	*Nepenthes gracilis*	Merckx,V.S. *et al.*	KP978679
240	*Nepenthes gracilis*	Merckx,V.S. *et al.*	KP978756
241	*Nepenthes gracilis*	Merckx,V.S. *et al.*	KP978745
242	*Nepenthes gracilis*	Merckx,V.S. *et al.*	KP978729
243	*Nepenthes gracilis*	Merckx,V.S. *et al.*	KP978718
244	*Nepenthes gracilis*	Merckx,V.S. *et al.*	KP978712
245	*Nepenthes gracilis*	Merckx,V.S. *et al.*	KP978691
246	*Nepenthes gracilis*	Merckx,V.S. *et al.*	KP978688
247	*Nepenthes gracilis*	Merckx,V.S. *et al.*	KP978682
248	*Nepenthes hamiguitanensis*	Acil,R.Y. *et al.*	KJ676886
249	*Nepenthes khasiana*	Meimberg,H. *et al.*	AF204836
250	*Nepenthes lowii*	Merckx,V.S. *et al.*	KP978673
251	*Nepenthes lowii*	Merckx,V.S. *et al.*	KP978723
252	*Nepenthes lowii*	Merckx,V.S. *et al.*	KP978722
253	*Nepenthes lowii*	Merckx,V.S. *et al.*	KP978717
254	*Nepenthes macfarlanei*	Meimberg,H. *et al.*	AF204832
255	*Nepenthes madagascariensis*	Meimberg,H. *et al.*	AF204835
256	*Nepenthes mirabilis*	Merckx,V.S. *et al.*	KP978675
257	*Nepenthes mirabilis*	Merckx,V.S. *et al.*	KP978685
258	*Nepenthes mirabilis*	Merckx,V.S. *et al.*	KP978686
259	*Nepenthes mirabilis*	Merckx,V.S. *et al.*	KP978702
260	*Nepenthes mirabilis*	Merckx,V.S. *et al.*	KP978708
261	*Nepenthes mirabilis*	Merckx,V.S. *et al.*	KP978711
262	*Nepenthes mirabilis*	Merckx,V.S. *et al.*	KP978713

263	*Nepenthes mirabilis*	Merckx,V.S *et al.*	KP978727
264	*Nepenthes mirabilis*	Merckx,V.S *et al.*	KP978734
265	*Nepenthes mirabilis*	Merckx,V.S *et al.*	KP978737
266	*Nepenthes mirabilis*	Merckx,V.S *et al.*	KP978761
267	*Nepenthes merrilliana*	Acil,R.Y. *et al.*	KJ676887
268	*Nepenthes micramphora*	Acil,R.Y. *et al.*	KJ676888
269	*Nepenthes mindanaoensis*	Acil,R.Y. *et al.*	KJ676889
270	*Nepenthes mindanaoensis*	Acil,R.Y. *et al.*	KM393200
271	*Nepenthes peltata*	Acil,R.Y. *et al.*	KJ676890
272	*Nepenthes pervillei*	Meimberg,H. *et al.*	AF204837
273	*Nepenthes pulchra*	Acil,R.Y. *et al.*	KJ676891
274	*Nepenthes rajah*	Merckx,V.S *et al.*	KP978690
275	*Nepenthes saranganiensis*	Acil,R.Y. *et al.*	KJ676892
276	*Nepenthes surigaoensis*	Acil,R.Y. *et al.*	KJ676893
277	*Nepenthes tentaculata*	Merckx,V.S *et al.*	KP978674
278	*Nepenthes tentaculata*	Merckx,V.S *et al.*	KP978676
279	*Nepenthes tentaculata*	Merckx,V.S *et al.*	KP978678
280	*Nepenthes tentaculata*	Merckx,V.S *et al.*	KP978680
281	*Nepenthes tentaculata*	Merckx,V.S *et al.*	KP978684
282	*Nepenthes tentaculata*	Merckx,V.S *et al.*	KP978692
283	*Nepenthes tentaculata*	Merckx,V.S *et al.*	KP978695
284	*Nepenthes tentaculata*	Merckx,V.S *et al.*	KP978698
285	*Nepenthes tentaculata*	Merckx,V.S *et al.*	KP978706
286	*Nepenthes tentaculata*	Merckx,V.S *et al.*	KP978709
287	*Nepenthes tentaculata*	Merckx,V.S *et al.*	KP978710
288	*Nepenthes tentaculata*	Merckx,V.S *et al.*	KP978714
289	*Nepenthes tentaculata*	Merckx,V.S *et al.*	KP978716
290	*Nepenthes tentaculata*	Merckx,V.S *et al.*	KP978719
291	*Nepenthes tentaculata*	Merckx,V.S *et al.*	KP978724
292	*Nepenthes tentaculata*	Merckx,V.S *et al.*	KP978730
293	*Nepenthes tentaculata*	Merckx,V.S *et al.*	KP978738
294	*Nepenthes tentaculata*	Merckx,V.S *et al.*	KP978739
295	*Nepenthes tentaculata*	Merckx,V.S *et al.*	KP978744
296	*Nepenthes tentaculata*	Merckx,V.S *et al.*	KP978746
297	*Nepenthes tentaculata*	Merckx,V.S *et al.*	KP978748
298	*Nepenthes tentaculata*	Merckx,V.S *et al.*	KP978754
299	*Nepenthes tentaculata*	Merckx,V.S *et al.*	KP978758
300	*Nepenthes thorelii*	Meimberg,H. *et al.*	AF204831
301	*Nepenthes tobaica*	Meimberg,H. *et al.*	AF204829
302	*Nepenthes tomoriana*	Meimberg,H. *et al.*	AF204830
303	*Nepenthes truncate*	Acil,R.Y. *et al.*	KJ676894
304	*Nepenthes veitchii*	Meimberg,H. *et al.*	AF204828
305	*Nepenthes ventricosa*	Acil,R.Y. *et al.*	KJ676895
306	*Nepenthes ventricosa*	Meimberg,H *et al.*	AF204833
307	*Nepenthes villosa*	Merckx,V.S *et al.*	KP978681

308	*Nepenthes villosa*	Merckx,V.S *et al.*	KP978701
309	*Nepenthes villosa*	Merckx,V.S *et al.*	KP978726
310	*Nepenthes villosa*	Merckx,V.S *et al.*	KP978742
311	*Nepenthes villosa*	Merckx,V.S *et al.*	KP978750
312	*Nepenthes x harryana*	Merckx,V.S *et al.*	KP978757
313	*Nepenthes x hookeriana*	Merckx,V.S *et al.*	KP978715
314	*Nepenthes x kinabaluensis*	Merckx,V.S *et al.*	KP978704
315	*Nepenthes x kinabaluensis*	Merckx,V.S *et al.*	KP978733
316	*Nepenthes x kinabaluensis*	Merckx,V.S *et al.*	KP978755
